# Association of Added Sugars Intake with Micronutrient Adequacy in US Children and Adolescents: NHANES 2009–2014

**DOI:** 10.1093/cdn/nzz126

**Published:** 2019-11-07

**Authors:** Victor L Fulgoni, P Courtney Gaine, Maria O Scott, Laurie Ricciuto, Loretta DiFrancesco

**Affiliations:** 1 Nutrition Impact, LLC, Battle Creek, MI, USA; 2 The Sugar Association, Inc., Washington, DC, USA; 3 The Sugar Association, Inc., Toronto, Canada; 4 Source! Nutrition, Toronto, Canada

**Keywords:** added sugars, micronutrient intake, micronutrient adequacy, children, adolescents, NHANES

## Abstract

**Background:**

A concern about the excessive consumption of added sugars is the potential for micronutrient dilution, particularly in children and adolescents; however, the evidence is inconsistent.

**Objective:**

We examined the associations between added sugars intake and micronutrient adequacy in US children and adolescents using data from NHANES 2009–2014.

**Methods:**

Children and adolescents aged 2–18 (*n* = 7754), 2–8 (*n* = 3423), and 9–18 y (*n* = 4331) were assigned to deciles of added sugars intake based on the average of 2 d of dietary recall. Usual intake of micronutrients was determined using 2 dietary recalls and the National Cancer Institute method. Within each age group, regression analyses were used to assess the relationship between added sugars intake decile and percentage of the population below the estimated average requirements (EARs) for 17 micronutrients.

**Results:**

Deciles of added sugars intake (percentage of calories) ranged from <6.4 to >22.8 among children and adolescents aged 2–18 y, with a median intake of 13.3% of calories. Significant positive associations (*P* < 0.01) between added sugars intake and percentage of the population (aged 2–18 y) below the EAR were found only for calcium, magnesium, and vitamin D. These associations virtually disappeared after dropping the 2 highest and lowest deciles of intake, suggesting a threshold effect; intakes below approximately 19% of calories from added sugars were generally not associated with micronutrient inadequacy.

**Conclusions:**

As added sugars intake increased, there was a threshold above which an increase in the prevalence of inadequate intakes for calcium, magnesium, and vitamin D among US children and adolescents was observed. However, even at the lower deciles of added sugars, large percentages of the population were below the EAR for these nutrients, suggesting that adequate intakes of these nutrients are difficult to achieve independent of added sugars intake.

## Introduction

In the United States and abroad, recent dietary guidance has provided recommendations for limits on added sugars intake in response to concerns of the association of added sugars with overweight and obesity, as well as the potential for micronutrient dilution of the diet. The Institute of Medicine's 2005 DRI report on macronutrients concluded that there is insufficient evidence to set a tolerable upper intake level for total and added sugars; however, the scientific committee suggested a maximum daily intake of 25% of calories from added sugars due to concerns about reduced intakes of micronutrients (1, 2). The 2015–2020 Dietary Guidelines for Americans (DGA) recommended limiting added sugars to <10% of total daily caloric intake; this target is based on food pattern modeling that demonstrated the public health need to limit calories from added sugars to meet food group and nutrient needs within calorie limits (3). Similarly, the WHO suggests that <10% of total energy be from “free sugars” (4); free sugars are inclusive of both added sugars and the sugars naturally present in 100% fruit juice.

Recent reviews have suggested that under isocaloric conditions, sugars in and of themselves do not contribute to obesity (3, 5) and chronic disease (6), but can lead to obesity and related comorbidities when consumed in excess. Reviews of the association of added sugars with micronutrient dilution have not uniformly supported this effect, and this research is often hampered by methodological issues (7–9). Authors have observed lower micronutrient intakes at both extremes of added sugars intake and have found that caloric intake seems to be the main predictor of micronutrient inadequacy (8, 10, 11). Nevertheless, the micronutrient dilution theory seems intuitive in the United States, where the major dietary sources of added sugars among youth are relatively consistent across the range of intakes of added sugars and include sugar-sweetened beverages, sweetened bakery items such as cookies and brownies, and candy and confectionery (12). Furthermore, few Americans in NHANES 2009–2012 were within the 10% of calories from added sugars guideline (12); some argue that this DGA target is difficult to achieve within current dietary patterns, and that it may also be difficult to achieve micronutrient recommendations with limited consumption of added sugars given their role in palatability of certain nutrient-rich foods such as flavored milk and ready-to-eat cereals (RTECs) (13). Mandatory and voluntary fortification practices may, in part, buffer the dilutions for certain micronutrients (14).

One study published subsequent to the earlier reviews and using NHANES 2003–2006 data showed mixed results: micronutrient intakes were lower with increased added sugars intakes among US children and adolescents; however, inadequacies (vitamins A and/or E) were observed at both lower and higher added sugars intakes (≤15% and >25% of calories, respectively), and at the higher intakes, the main issue was one of overall high-calorie and low-quality diets (10). This previous study did not fully evaluate the impact of added sugars on intakes of calcium and vitamin D because it was conducted prior to the establishment of the Estimated Average Requirements (EARs) for these nutrients; it also set added sugars intakes at 5% increments. One other study (11) showed that added sugars intakes in children were associated with increased nutrient inadequacy only for calcium and vitamin E; however, these researchers categorized added sugars intake into finite intake groups (i.e., 0 to <5%, 5% to <10%, 10% to <15%, 15% to <20%, 20% to <25%, and ≥25% of energy as added sugars).

The purpose of the present study was to examine contemporary intakes of added sugars in US children and adolescents, and to examine the relationships between added sugars and usual dietary intakes and adequacy of vitamins and minerals using deciles of added sugars intake.

## Methods

### Data source and participants

NHANES is a nationally representative cross-sectional survey conducted by the National Center for Health Statistics, part of the US CDC. The study protocol was approved by the National Center for Health Statistics research ethics review board. Written informed consent was obtained for all participants directly or through proxy for children (15). An overview of NHANES, including the purpose, study population, sampling strategy, survey procedures, and response rates is provided elsewhere (16).

Briefly, dietary intake data were collected as part of What We Eat in America (the dietary intake interview component of the NHANES) by two 24-h dietary recalls, the first collected in person during the health examination and the second conducted via telephone 3–10 d after the first. Both 24-h recalls were collected using the USDA Automated Multiple-Pass Method (17, 18). Caretakers provided the 24-h dietary recall information for children aged 2–5 y; children aged 6–11 y were assisted by an adult, and older children and adolescents provided their own recall information. For our analysis, we combined data from NHANES 2009–2014 from 8583 children and adolescents aged 2–18 y, excluding those with unreliable dietary records as determined the USDA National Center for Health Statistics staff (*n* = 99), those with zero calories (*n* = 2), and those with only a single dietary recall (*n* = 1325). The final sample included 7754 children (3927 males and 3827 females), with 3423 (1764 males and 1659 females) participants aged 2–8 y and 4331 (2163 males and 2168 females) participants aged 9–18 y. NHANES data on intakes of added sugars among children younger than 2 y have been previously reported, and this age group was therefore excluded from this analysis (19).

### Added sugars and micronutrient intakes

Added sugars intake was determined using the USDA Food Patterns Equivalent Database (FPED) for each NHANES release (20). The FPED defines added sugars as “sugars that are added to foods as an ingredient during preparation, processing, or at the table.” Added sugars do not include naturally occurring sugars such as lactose present in milk, fructose present in whole or cut fruit, and 100% fruit juice; for FPED 2011–2012 and 2013–2014, but not FPED 2009–2010, fruit juice concentrates used as ingredients are also assigned to added sugars (20). Data regarding intakes of added sugars in grams and calories were outputted, and percentages of total calories from added sugars were determined. Subjects were then placed into deciles of added sugars intake as percentage of total calories based on their average added sugars intakes (as percentage of calories for both days) over both 24-h dietary recalls; separate deciles were established for each age group (2–18, 2–8, and 9–18 y). Using the average of 2 d of intake to define added sugars intake as a percentage of calories in contrast to a single dietary recall or usual intakes was chosen to allow direct comparison to previously published work (10).

Intakes of 17 micronutrients with an EAR were obtained from NHANES dietary intake files; these were calcium, copper, iron, magnesium, phosphorus, selenium, and zinc, and folate, niacin, riboflavin, thiamin, and vitamins A, B_6_, B_12_, C, D, and E. The National Cancer Institute (NCI) Method was used to estimate usual intake (UI) of nutrients and their respective distribution of intake (21) in the 2–18, 2–8, and 9–18 y age groups. Given most micronutrients were consumed on most days by most subjects, the one part model was used for the UI estimations. The 2 d of intake, using day one sampling weights, were used to obtain percentiles of intake and necessary variance estimates. Covariates used in the NCI UI estimations were day of the week of the 24-h recall [coded as weekend (Friday to Sunday) or weekday (Monday to Thursday)] and sequence of dietary recall (first or second). Balanced repeated replication was performed to generate standard errors and balanced repeated replication weights were generated using Fay adjustment factor M = 0.3 with perturbation factor of 0.7, and then adjusted to match initial sample weights within age, gender, and race/ethnicity groups. Given the EAR is the appropriate DRI to use when assessing the adequacy of population micronutrient intakes (22), the EAR cut-point method was used to estimate the percentage of an age group with intakes below requirements (prevalence of inadequacy) within each decile of added sugars intake. However, to determine the prevalence of inadequate intake of iron, the probability method was used (22). To determine the percentage of the population below the EAR when the EAR was different based on age/gender, we divided each subject's intake in the Monte Carlo dataset generated by the NCI program by the subject specific EAR and then used the percentage less than the fixed value of 1.0. Micronutrient contributions from dietary supplements were not included.

### Statistical analysis

Linear regression analyses were conducted within the 3 age groups (2–18, 2–8, and 9–18 y) to determine whether relationships existed between decile of added sugars intake and micronutrient inadequacy. Dummy-coded deciles of intake (1–10)were used as the independent variable. Regression coefficients were generated to examine the magnitude of significant relationships and showed the change in percentage of the population with inadequate intakes (below the EAR) as added sugars intake increased. Sensitivity analysis was performed to examine the influence of the extremes of intakes of added sugars to the main findings, first by restricting the analysis to deciles 2 through 9, and second by restricting the analysis to deciles 3 through 8. Additionally, to assess whether the relationship of added sugars intake with prevalence of nutrient inadequacy was curvilinear, regression analyses were conducted using the linear and quadratic term of dummy variable for decile number; if the quadratic term was significant then a curvilinear relationship was present. Finally, we also generated linear regression results, adjusting for the average energy intakes of the deciles. Statistical significance was set at *P* < 0.01, and *t* tests were used to assess whether the regression coefficient was significantly different from zero.

## Results

### Added sugars intakes

The deciles of added sugars intake (percentage of calories) using the average intake over a 2-d period ranged from <6.4 to >22.8 for children and adolescents 2–18 y (**Table 1**). Median added sugars intake was 13.3% of calories for the population aged 2–18 y and was lower (12.2%) among children aged 2–8 y and higher (14.1%) among those aged 9–18 y. Based on the 2 dietary recalls for these age populations, the mean ± SE percentages of those aged 2–18 y who met the DGA recommendation to consume <10% of calories daily from added sugars (4) were 28.9% ± 1.0%, whereas of those aged 2–8 and 9–18 y, 32.3% ± 1.0% and 26.0% ± 1.3%, respectively, had intakes below this limit. The percentages of the populations with intakes of <5% of calories from added sugars were 5.5% ± 0.5%, 5.6% ± 0.6%, and 5.4% ± 0.7% of children and adolescents aged 2–18, 2–8, and 9–18 y, respectively.

**TABLE 1:** Deciles of added sugar intake (percentage of calories) of children and adolescents aged 2–18 y (NHANES 2009–2014)^1^

	Age group		
Decile	2–18 y (*n* = 7754)	2–8 y (*n* = 3423)	9–18 y (*n* = 4331)
1	<6.4	<6.1	<6.7
2	≥6.4 to ≤8.5	≥6.1 to ≤8.0	≥6.7 to ≤9.0
3	>8.5 to ≤10.2	>8.0 to ≤9.6	>9.0 to ≤10.8
4	>10.2 to ≤11.6	>9.6 to ≤10.7	>10.8 to ≤12.4
5	>11.6 to ≤13.3	>10.7 to ≤12.2	>12.4 to ≤14.1
6	>13.3 to ≤15.0	>12.2 to ≤13.8	>14.1 to ≤15.9
7	>15.0 to ≤16.7	>13.8 to ≤15.5	>15.9 to ≤17.8
8	>16.7 to ≤19.1	>15.5 to ≤17.4	>17.8 to ≤20.3
9	>19.1 to ≤22.8	>17.4 ≤ 20.6	>20.3 to ≤24.6
10	>22.8	>20.6	>24.6

1 Using the average of percentage of calories from added sugars from 2 d of dietary recall. Data source for added sugars: USDA (20) Food Patterns Equivalent Database 2009–2010, 2011–2012, and 2013–2014.

### Relationship of added sugars intake to micronutrient adequacy

For children and adolescents aged 2–18 y, there was a significant association between the deciles of added sugars intakes and micronutrient adequacies for 3 of the 17 nutrients that were examined, calcium (*β* ± SE: 3.1 ± 0.5), magnesium (1.9 ± 0.4), and vitamin D (1.8 ± 0.2) (**Table 2** and **Supplemental Tables 1–3**). This result for calcium indicated that each 1-decile increase in added sugars intake was associated with an increase of 3.1% of children and adolescents aged 2–18 y with inadequate calcium intakes (below the EAR). The association with calcium was also significant among children aged 2–8 y (1.7 ± 0.4) and children and adolescents aged 9–18 y (4.0 ± 0.7). For magnesium, the association between added sugars intake and percentage below the EAR was significant among children and adolescents aged 2–18 and 9–18 y but did not reach significance among children aged 2–8 y; each 1-decile increase in added sugars intake was associated with an increase of 1.9% of children and adolescents aged 2–18 y with inadequate magnesium intakes. For vitamin D, associations were significant in all age subgroups, with each 1-decile increase in added sugars intake associated with an increase of 1.8% of those aged 2–18 y with inadequate vitamin D intakes. Additionally, there was a significant association for vitamin E among children and adolescents aged 9–18 y; each 1-decile increase in added sugars intake was associated with an increase of 1.7% of those with inadequate vitamin E intakes.

**TABLE 2:** Association between added sugars intake (% of calories) and percentage of children and adolescents 2–18 y (NHANES 2009–2014) with micronutrient intakes below the EAR^1^

	Age group					
	2–18 y (*n* = 7754)		2–8 y (*n* = 3423)		9–18 y (*n* = 4331)	
Nutrient	*β* ^2^ ± SE	*P* ^3^	*β* ± SE	*P*	*β* ± SE	*P*
Minerals						
Calcium	3.07 ± 0.54	0.0004*	1.72 ± 0.38	0.0020*	3.97 ± 0.71	0.0005*
Copper	0.20 ± 0.28	0.4949	−0.01 ± 0.01	0.4487	0.44 ± 0.46	0.3733
Iron	0.17 ± 0.12	0.1740	0.02 ± 0.04	0.6032	0.27 ± 0.19	0.1972
Magnesium	1.93 ± 0.42	0.0017*	0.06 ± 0.06	0.4040	3.67 ± 0.65	0.0005*
Phosphorus	0.95 ± 0.54	0.1142	0.004 ± 0.008	0.6246	1.43 ± 0.85	0.1314
Selenium	−0.01 ± 0.01	0.3137	0.00002 ± 0.0002	0.9280	−0.01 ± 0.01	0.4365
Zinc	0.57 ± 0.52	0.3024	0.02 ± 0.05	0.7119	1.03 ± 0.85	0.2591
Vitamins						
Folate	0.40 ± 0.19	0.0656	0.01 ± 0.01	0.4798	0.68 ± 0.29	0.0460
Niacin	0.003 ± 0.03	0.9241	0.004 ± 0.007	0.4960	0.01 ± 0.05	0.9210
Riboflavin	0.07 ± 0.09	0.4473	0.004 ± 0.004	0.3874	0.16 ± 0.14	0.2670
Thiamin	0.12 ± 0.11	0.3022	0.01 ± 0.006	0.1139	0.20 ± 0.18	0.3025
Vitamin A	1.95 ± 0.61	0.0126	0.19 ± 0.16	0.2519	2.82 ± 0.94	0.0171
Vitamin B_12_	−0.01 ± 0.08	0.9037	−0.003 ± 0.006	0.6894	−0.03 ± 0.14	0.8217
Vitamin B_6_	0.08 ± 0.11	0.4761	0.0007 ± 0.008	0.9335	0.15 ± 0.19	0.4498
Vitamin C	0.98 ± 0.48	0.0752	0.05 ± 0.11	0.6573	1.51 ± 0.74	0.0734
Vitamin D	1.76 ± 0.22	<0.0001*	2.20 ± 0.34	0.0002*	1.56 ± 0.17	<0.0001*
Vitamin E	1.00 ± 0.35	0.0207	0.07 ± 0.50	0.8971	1.74 ± 0.39	0.0021*

1 EAR, Estimated Average Requirement.

2 Regression coefficient (*β*) of association of percentage of the population below the EAR as assessed with usual intakes across deciles of added sugars intake as defined as the average of the percentage of calories from added sugars using 2 d of dietary recall.

3 *P* value for estimating the probability of rejecting the null hypothesis that the slope of the association is zero (*β* = 0) when it is true; *P < 0.01 deemed significant.

When linear regression analyses were repeated with the lowest and highest deciles dropped (1 and 10), the positive associations between added sugars intake and percentage of the population (children and adolescents aged 2–18 y) below the EAR persisted for calcium, magnesium, and vitamin D; while inadequacy of folate and vitamin A was also significantly related to added sugars intake (**Table 3**). Among children and adolescents aged 2–18 y, the percentage with calcium intakes below the EAR increased from 36% to 61% from deciles 2 to 9, and the percentage below the EAR for magnesium increased from 31% to 40%; lower increases of percentages below the EARs for vitamin D and folate were 76% to 94% (24% increase) and 3.5% to 4.4% (26% increase), respectively. When the 2 highest and the 2 lowest deciles were dropped (deciles 1 and 2 and 9 and 10), there was no association of added sugars intake with any of the 17 micronutrients among children and adolescents aged 2–18 y (for intake ranges of 8.5% to 19.1% of total calories) and children aged 2–8 y (for intake ranges of 8% to 17.4% of total calories); only the association between added sugars intake and percentage of the population below the EAR for magnesium in those aged 9–18 y persisted (**Table 4**).

**TABLE 3:** Association between added sugars intake (% of calories) and percentage of children and adolescents 2–18 y (NHANES 2009–2014) with micronutrient intakes below the EAR for added sugars intake deciles 2 through 9^1^

	Age group					
	2–18 y (*n* = 7754)		2–8 y (*n* = 3423)		9–18 y (*n* = 4331)	
Nutrient	*β* ^2^ ± SE	*P* ^3^	*β* ± SE	*P*	*β* ± SE	*P*
Minerals						
Calcium	2.71 ± 0.51	0.0018*	1.80 ± 0.50	0.0117	3.27 ± 0.64	0.0022*
Copper	0.23 ± 0.19	0.2658	−0.004 ± 0.01	0.5212	0.46 ± 0.32	0.2043
Iron	0.19 ± 0.07	0.0310	0.01 ± 0.05	0.7928	0.31 ± 0.11	0.0301
Magnesium	1.63 ± 0.27	0.0009*	0.06 ± 0.05	0.3086	2.92 ± 0.42	0.0005*
Phosphorus	0.90 ± 0.55	0.1513	0.004 ± 0.007	0.5740	1.39 ± 0.0.85	0.1554
Selenium	−0.01 ± 0.01	0.5339	0.00003 ± 0.0001	0.7560	−0.004 ± 0.012	0.7441
Zinc	0.49 ± 0.53	0.3910	0.03 ± 0.06	0.6313	0.86 ± 0.89	0.3715
Vitamins						
Folate	0.40 ± 0.10	0.0067*	0.01 ± 0.01	0.4083	0.70 ± 0.14	0.0024*
Niacin	0.20 ± 03	0.4510	0.007 ± 0.005	0.2175	0.02 ± 0.04	0.6577
Riboflavin	0.09 ± 0.06	0.2008	0.004 ± 0.003	0.3527	0.20 ± 0.08	0.0480
Thiamin	0.15 ± 0.09	0.1323	0.011 ± 0.005	0.0549	0.30 ± 0.14	0.0714
Vitamin A	1.71 ± 0.33	0.0020*	0.19 ± 0.15	0.2536	2.63 ± 0.50	0.0019*
Vitamin B_12_	0.02 ± 0.09	0.8063	−0.003 ± 0.006	0.6702	0.07 ± 0.15	0.6658
Vitamin B_6_	0.11 ± 0.07	0.1565	0.004 ± 0.006	0.6636	0.23 ± 0.12	0.1158
Vitamin C	0.47 ± 0.25	0.1049	0.02 ± 0.14	0.9048	0.85 ± 0.46	0.1158
Vitamin D	1.62 ± 0.32	0.0021*	1.85 ± 0.30	0.0008*	1.46 ± 0.33	0.0044*
Vitamin E	0.89 ± 0.51	0.1293	0.67 ± 0.71	0.2810	1.11 ± 0.57	0.1003

1 EAR, Estimated Average Requirement.

2 Regression coefficient (*β*) of association of percentage of the population below the EAR as assessed with usual intakes across deciles of added sugars intake as defined as the average of the percentage of calories from added sugars using 2 d of dietary recall.

3 *P* value for estimating the probability of rejecting the null hypothesis that the slope of the association is zero (*β* = 0) when it is true; *P < 0.01 deemed significant.

**TABLE 4:** Association between added sugars intake (% of calories) and percentage of children and adolescents 2–18 y (NHANES 2009–2014) with micronutrient intakes below the EAR for added sugars intake deciles 3 through 8^1^

	Age group					
	2–18 y (*n* = 7754)		2–8 y (*n* = 3423)		9–18 y (*n* = 4331)	
Nutrient	*β* ^2^ ± SE	*P* ^3^	*β* ± SE	*P*	*β* ± SE	*P*
Minerals						
Calcium	2.87 ± 0.98	0.0432	2.42 ± 0.84	0.0453	2.93 ± 1.17	0.0656
Copper	0.14 ± 0.28	0.6444	−0.01 ± 0.01	0.4041	0.17 ± 0.49	0.7499
Iron	0.10 ± 0.11	0.4119	−0.01 ± 0.10	0.9342	0.23 ± 0.17	0.2361
Magnesium	1.56 ± 0.38	0.0151	0.10 ± 0.07	0.2200	2.80 ± 0.61	0.0099*
Phosphorus	0.83 ± 0.70	0.3011	0.004 ± 0.009	0.6413	1.29 ± 0.1.09	0.3017
Selenium	−0.01 ± 0.01	0.3550	0.00003 ± 0.0001	0.8066	−0.01 ± 0.02	0.6605
Zinc	0.65 ± 0.71	0.4081	0.02 ± 0.08	0.8253	0.93 ± 1.24	0.4936
Vitamins						
Folate	0.45 ± 0.19	0.0727	0.02 ± 0.02	0.2398	0.69 ± 0.29	0.0779
Niacin	0.04 ± 0.04	0.3361	0.01 ± 0.01	0.2419	0.04 ± 0.06	0.5480
Riboflavin	0.09 ± 0.08	0.3374	0.003 ± 0.004	0.4932	0.20 ± 0.10	0.1021
Thiamin	0.21 ± 0.12	0.1687	0.01 ± 0.01	0.1720	0.38 ± 0.19	0.1158
Vitamin A	1.95 ± 0.52	0.0199	0.27 ± 0.22	0.2845	3.05 ± 0.77	0.0166
Vitamin B_12_	0.05 ± 0.17	0.7578	0.003 ± 0.015	0.8647	0.10 ± 0.26	0.7276
Vitamin B_6_	0.06 ± 0.12	0.6291	0.004 ± 0.008	0.6684	0.13 ± 0.22	0.5754
Vitamin C	0.80 ± 0.38	0.1049	0.14 ± 0.27	0.6281	1.28 ± 0.73	0.1557
Vitamin D	1.39 ± 0.65	0.1002	2.02 ± 0.63	0.0324	1.11 ± 0.67	0.1732
Vitamin E	0.46 ± 0.72	0.5606	0.27 ± 1.31	0.8495	0.38 ± 0.66	0.5923

1 EAR, Estimated Average Requirement.

2 Regression coefficient (*β*) of association of percentage of the population below the EAR as assessed with usual intakes across deciles of added sugars intake as defined as the average of the percentage of calories from added sugars using 2 d of dietary recall.

3 *P* value for estimating the probability of rejecting the null hypothesis that the slope of the association is zero (*β* = 0) when it is true; *P < 0.01 deemed significant.

**FIGURE 1:**
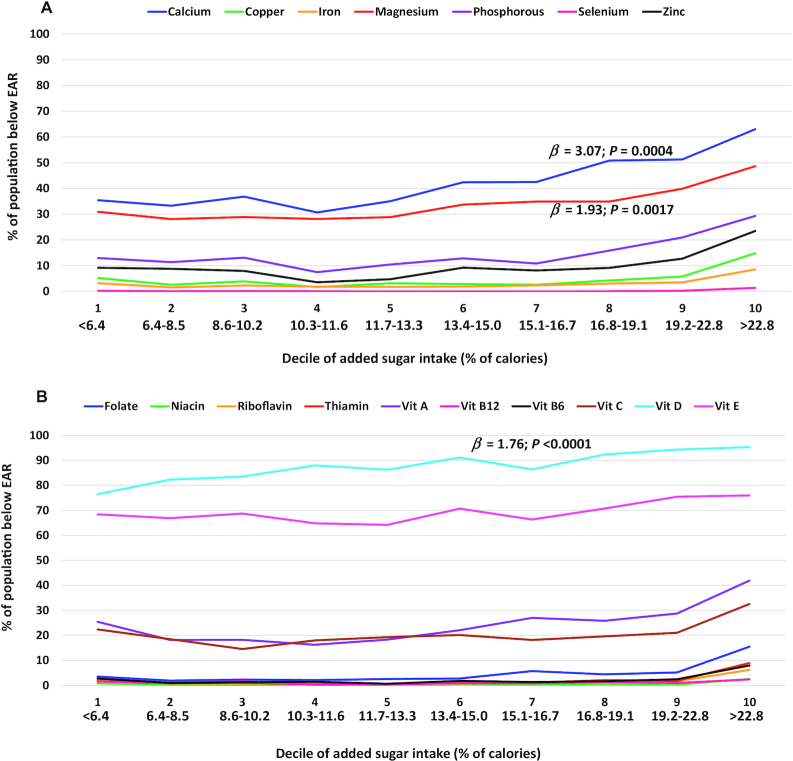
Percentage of children and adolescents aged 2–18 y (NHANES 2009–2014) with micronutrient intakes below the EARs for (A) minerals and (B) vitamins per decile of added sugars intake (percentage of calories) averaged across 2 d of dietary recall using regression analysis and regression coefficient (*β*). Association deemed significant at *P* < 0.01. EAR, Estimated Average Requirement; Vit, vitamin.

**FIGURE 2:**
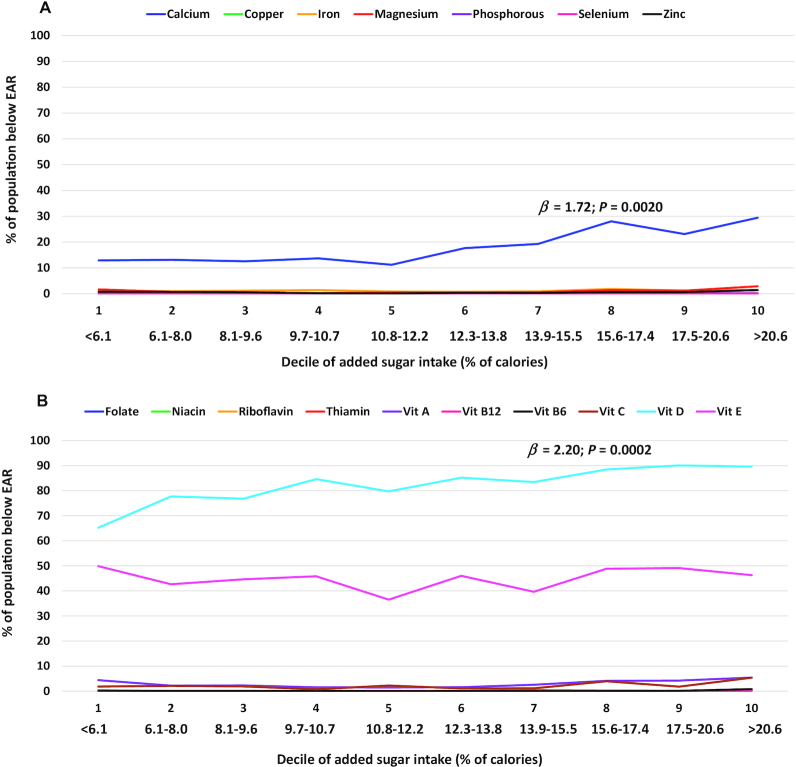
Percentage of children aged 2–8 y (NHANES 2009–2014) with micronutrient intakes below the EARs for (A) minerals and (B) vitamins per decile of added sugars intake (percentage of calories) averaged across 2 d of dietary recall using regression analysis and regression coefficient (*β*). Association deemed significant at *P* < 0.01. EAR, Estimated Average Requirement; Vit, vitamin.

**FIGURE 3:**
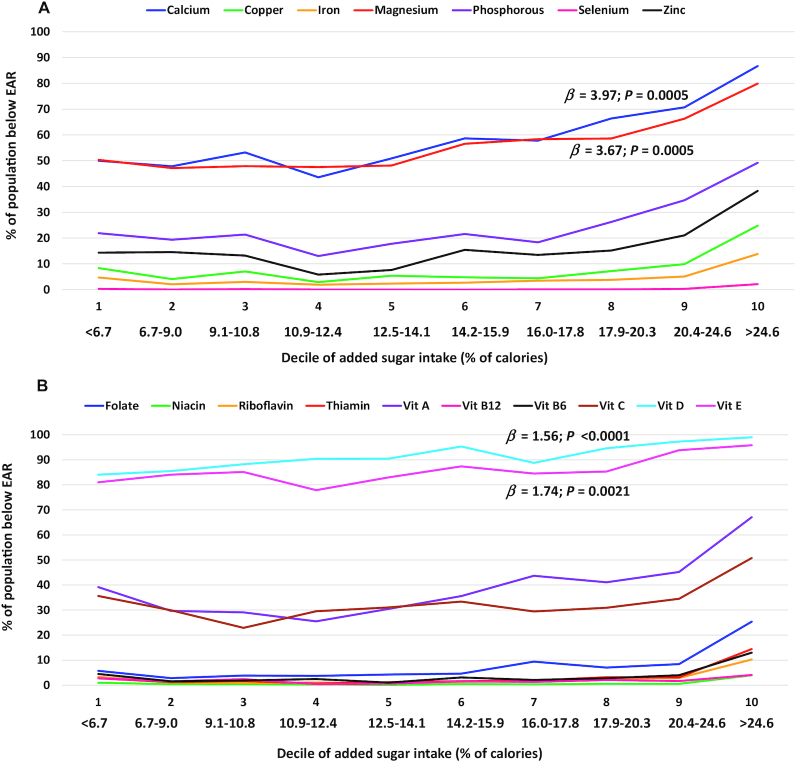
Percentage of children and adolescents aged 9–18 y (NHANES 2009–2014) with micronutrient intakes below the EARs for (A) minerals and (B) vitamins per decile of added sugars intake (percentage of calories) averaged across 2 d of dietary recall using regression analysis and regression coefficient (*β*). Association deemed significant at *P* < 0.01. EAR, Estimated Average Requirement; Vit, vitamin.

For the micronutrients with significant overall associations with added sugars, results of the linear and quadratic regression analyses indicated curvilinear relationships between added sugars intake and micronutrient inadequacy for calcium and magnesium among children and adolescents aged 2–18 y; while for vitamin D, the linear term was significant, but not the quadratic term. Among children aged 2–8 y, only linear terms were significant for calcium and vitamin D; while among those children and adolescents aged 9–18 y, curvilinear relationships between added sugars intake and micronutrient adequacy were observed for calcium and magnesium, but not for vitamin D. Additionally, a curvilinear relationship was observed for vitamin E among those aged 9–18 y. The *β* coefficient results from these quadratic regression analyses are not shown; however, the curvilinear nature of the relationships for certain nutrients can be observed from the presentation of our results from the linear regressions across the added sugars deciles (**Figures 1**–**3**).

The impact on linear regression results of adjusting for energy intake in deciles of added sugars intake depended on the age group. In those children and adolescents aged 2–18 y (and similarly those aged 9–18 y), after energy adjustment the adequacies of copper, phosphorus, zinc, vitamin A, and vitamin C were also found to be associated with deciles of added sugars intake (**Table 5**), whereas in children aged 2–8 y, there was less impact after energy adjustment; calcium and vitamin D inadequacies remained significantly associated with added sugars intake and only iron was also significantly associated with added sugars intake (Table 5), albeit with a small increase of 0.1% of the population with iron inadequacy for each decile change in added sugars.

**TABLE 5:** Association between added sugars intake (% of calories) and percentage of children and adolescents 2–18 y (NHANES 2009–2014) with micronutrient intakes below the EAR^1^ after adjustment for energy intake

	Age group					
Nutrient	2–18 y (*n* = 7754)		2–8 y (*n* = 3423)		9–18 y (*n* = 4331)	
Minerals	*β* ^2^ ± SE	*P* ^3^	*β* ± SE	*P*	*β*± SE	*P*
Calcium	4.81 ± 0.63	0.0001*	2.82 ± 0.49	0.0007*	4.48 ± 0.37	<0.0001*
Copper	1.27 ± 0.41	0.0175	−0.006 ± 0.013	0.6806	1.23 ± 0.35	0.0095*
Iron	0.69 ± 0.22	0.0171	0.14 ± 0.03	0.0051*	0.54 ± 0.20	0.0328
Magnesium	3.44 ± 0.42	0.0001*	0.10 ± 0.12	0.3984	3.80 ± 0.36	<0.0001*
Phosphorus	4.32 ± 0.74	0.0006*	0.01 ± 0.01	0.3925	3.25 ± 0.42	0.0001*
Selenium	0.02 ± 0.02	0.4509	0.00001 ± 0.0002	0.9488	−0.01 ± 0.02	0.4962
Zinc	2.56 ± 0.65	0.0059*	0.10 ± 0.06	0.1571	2.38 ± 0.58	0.0046*
Vitamins						
Folate	1.30 ± 0.38	0.0116	0.03 ± 0.01	0.0817	1.03 ± 0.39	0.0336
Niacin	0.07 ± 0.07	0.3497	0.02 ± 0.01	0.0482	0.05 ± 0.08	0.5749
Riboflavin	0.30 ± 0.17	0.1216	0.011 ± 0.004	0.0393	0.26 ± 0.17	0.1679
Thiamin	0.42 ± 0.15	0.0286	0.023 ± 0.009	0.0349	0.42 ± 0.15	0.0286
Vitamin A	3.63 ± 0.73	0.0016*	0.49 ± 0.18	0.0309	3.75 ± 0.77	0.0018*
Vitamin B_12_	0.33 ± 0.09	0.0088*	0.002 ± 0.007	0.7732	0.13 ± 0.13	0.3471
Vitamin B_6_	0.40 ± 0.26	0.1758	0.016 ± 0.009	0.1143	0.40 ± 0.27	0.1731
Vitamin C	2.42 ± 0.68	0.0092*	0.19 ± 0.24	0.4482	2.11 ± 0.52	0.0049*
Vitamin D	1.53 ± 0.30	0.0015*	1.97 ± 0.52	0.0069*	1.50 ± 0.19	0.0001*
Vitamin E	1.76 ± 0.50	0.0099*	1.37 ± 0.58	0.0489	1.62 ± 0.17	<0.0001*

1 EAR, Estimated Average Requirement.

2 Regression coefficient (*β*) of association of percentage of the population below the EAR as assessed with usual intakes across deciles of added sugars intake as defined as the average of the percentage of calories from added sugars using 2 d of dietary recall after adjustment for energy intake.

3 *P*-value for estimating the probability of rejecting the null hypothesis that the slope of the association is zero (*β* = 0) when it is true; *P < 0.01 deemed significant.

## Discussion

Our results across deciles of added sugars intake in US children and adolescents determined using data from NHANES 2009–2014 provide recent estimates of added sugars intakes for this population. Median intake of added sugars based on the average of 2 d of dietary recall was 13.3% of calories among children and adolescents 2–18 y. Compared with the results of an earlier analysis using NHANES 2011–2012 data, which reported a mean added sugars intake of 17% of calories (23), our results suggest a slightly lower intake of added sugars among US children and adolescents. The degree to which the increased public health focus on added sugars, and subsequent changes in consumer behavior and food product development or reformulation in response to this focus, have contributed to lower intakes warrants further study.

Of the 17 micronutrients we examined, the USDA has reported that at least 1 of the 8 age/gendergroups (1–3, 4–8, 9–13, and 14–18 y) evaluated had at least 25% of the population below the EARs for calcium, magnesium, phosphorus, and vitamins A, C, D, and E (24). We found significant associations between added sugars intake and percentage of the population below the EAR for only 3 nutrients: calcium, magnesium, and vitamin D. Our findings are consistent with those reported in previous studies on added sugars intake in North America, in which intakes of calcium, magnesium, vitamin D, and vitamin C were identified as having significant relationships with added sugars intakes among children and adolescents (11, 13, 25, 26). Other studies have shown significant relationships with added sugars intake and vitamins A and E, iron, and folate (6, 7, 13, 25). While our data did not show significant relationships of added sugars intakes with these nutrients, this discrepancy may be due to differences in current fortification practices, as these nutrients are routinely added to foods, especially RTEC, or to different analytical methodologies used across studies. Further monitoring of the impact of added sugars on nutrient intakes is warranted, especially as food trends continue to evolve.

The types of foods and beverages containing added sugars that are consumed at different levels of added sugars intake may partly explain the associations we found. A recent study showed that among children and adolescents aged 2–18 y, top sources of added sugars among those consuming the lowest amount of added sugars (decile 1) were RTEC, sweet bakery products, and sweetened beverages, which together accounted for 33% to 43% of added sugars intake, depending on the age group (12). Among those consuming the highest amount of added sugars (decile 10), the sweetened beverages category was the top contributor, accounting for 37% to 53% of added sugars intake, depending on the age group (12). The lack of nutrients in sweetened beverages may explain the higher micronutrient inadequacies apparent across higher deciles of added sugars intake, whereas nutrients provided by RTEC and the milk (calcium, vitamin D, magnesium) that tends to be consumed with them may contribute to the lower micronutrient inadequacies that we observed across lower deciles of added sugars intake. This explanation is supported by other research showing that the consumption of RTEC increased the likelihood of children and adolescents aged 6–17 y meeting recommendations for iron and folate (27). These findings suggest that essential nutrients in foods containing added sugars can make important contributions to micronutrient adequacy, and thus that knowledge of the entire nutrient composition of foods, and not only 1 component, such as added sugars, will be more helpful to informing dietary guidance.

Our additional analyses, for which we dropped low and high deciles of added sugars intake, helped us to assess a threshold association of added sugars intake with micronutrient inadequacy. As the decile of added sugars intake increased, there was an increase in the percentage of the population below the EARs for calcium, magnesium, and vitamin D, which persisted when we eliminated the lowest and the highest deciles, but which virtually disappeared (only magnesium inadequacy remained significant in 1 age group) when the top 2 and the bottom 2 deciles were dropped from the analyses. Given that the high end of the second highest decile (decile 9) was <19.1% of calories from added sugars, there appears to be an intake threshold, whereby added sugars intakes above this level more dramatically increase inadequacies of calcium, magnesium, and vitamin D. This interpretation is consistent with other research showing a threshold effect, whereby nutrient inadequacies increased significantly only above a certain level of added sugars intake (20% to 25% of total calories) (10). The shift to greater consumption of sweetened beverages among the highest consumers of added sugars might contribute to this threshold effect, insofar as the beverages replace sources of added sugars that also provide essential micronutrients (such as RTEC and sweet bakery products) (12).

Analyses of regression results adjusting for energy intake indicated some impact depending on the age group, with more of an impact in those aged 9–18 y and little impact in those aged 2–8 y. While the range in calories across the deciles was similar [227 kcal (1878–2105 kcal) in those 9–18 y compared with 246 kcal (1521 to 1767 kcal) in those 2–8 y], more nutrients had a significant regression coefficient for energy intake in those aged 9–18 y. This finding suggests that the quality of the diet of children and adolescents aged 9–18 y was less than that of children aged 2–8 y, and that added sugars intake had a larger impact on micronutrient adequacy in the older age group, in which EAR levels increased for many nutrients. These results should not be surprising, because if energy is held constant and amounts of added sugars increases, then nutrient intakes are likely to decrease. These results show the theoretical impact of added sugars intake on nutrient adequacy; what is interesting is that even in these theoretical situations, micronutrient adequacy of many nutrients was still not associated with added sugars intake, which is likely due to the selection of some nutrient-dense and fortified foods that contain added sugars.

In the US population, calcium and vitamin D are characterized as nutrients of public health concern because low intakes are associated with adverse health effects; while magnesium is considered a shortfall nutrient (28). Although we observed an increase in the prevalence of inadequacy for these micronutrients as added sugars intake increased, substantial percentages of the population were below the EAR, particularly for vitamin D, even at lower deciles of added sugars intake. Low intakes of key food groups, including dairy, vegetables, and whole grains, that are important sources of these nutrients of concern have been reported among children and adolescents regardless of added sugars intake (4), lending support to our findings of inadequacies across the full range of added sugars intakes. Taken together, these results suggest that adequate intakes of calcium, magnesium, and vitamin D are difficult for US children and adolescents to achieve given their current dietary patterns, independent of added sugars intake. Additionally, in those children and adolescents aged 2–18 y, for certain nutrients (vitamins A and C) the lowest decile of added sugars intake was associated with higher micronutrient inadequacy levels than the next several deciles of added sugars intake (Supplemental Table 1). Others have reported that low intakes of added sugars (<10% of calories) were not necessarily associated with greater intake of micronutrients (29).

The quality of the diet of children and adolescents could be improved by increased consumption of foods and beverages that are sources of these nutrients of concern and other shortfall nutrients. An analysis of NHANES 2009–2010 data showed that adding a daily serving of yogurt to diets with insufficient amounts of calcium would provide enough calcium for children aged 9–11 y to achieve adequate intake and would increase vitamin D consumption of children aged 2–11 y (30). In girls and women aged 9–20 y, inadequate intakes of calcium, magnesium, and vitamin C were improved with higher dairy and fruit and vegetable consumption (31). Particular attention could be given to shifting consumption patterns away from nutrient poor, sweetened beverages among heavy consumers of these products toward products containing essential nutrients that will help children and adolescents meet their micronutrient requirements. Further research specifically examining the impacts of added sugars in those children meeting key food recommendations (such as numbers of whole grain servings, fruit/vegetable servings, and/or dairy servings) would be helpful to understand the role these foods play in mitigating micronutrient inadequacies in the presence of added sugars.

Our study has some strengths and limitations. Using NHANES data from 3 cycles provided a large amount of data collected under commonly accepted procedures and allowed for more up-to-date estimates of added sugars intakes among US children and adolescents; and averaging added sugars intakes across 2 dietary recalls provided a better estimate of intake than using a single recall. A key strength of our analyses was using both days of intake to determine usual micronutrient intake and the distribution of nutrient intake to assess percentage of the population below the EAR with the NCI method. Regression analyses allowed us to estimate the magnitude of change in the percentage of the population below the EAR as added sugars increased. Furthermore, we used deciles of added sugars intake based on the average of the 2 d of dietary recall versus arbitrarily selected set points, allowing us to examine points along the continuum of added sugars intake where micronutrient intakes are sufficient and/or begin to be a concern.

Limitations of this study include the use of dietary intake data that are self-reported, in some cases from caregivers; while the best procedures were used to assess micronutrient intake, self-reported dietary data rely on memory and the ability of subjects or caregivers to accurately report the foods and amounts of foods consumed. It is also unknown if there is an under-reporting bias regarding foods with added sugars; if such bias exists, it would impact our added sugars intake estimates and possibly lead to misclassification of subjects to deciles of intake. Additionally, any type of categorization (assignment to added sugars deciles) is subject to misclassification error, which can make finding significant regression results less likely.

In conclusion, using NHANES 2009–2014 data, our study demonstrated significant associations between added sugars intake and micronutrient adequacy among US children and adolescents aged 2–18 y for 3 of 17 micronutrients examined. Using deciles as the added sugars intake variable, rather than arbitrary cutoff points, we were able to observe trends in micronutrient adequacy along the continuum of added sugars intake. As decile of added sugars intake increased, there was an increase in the percentage of the population below the EARs for calcium, magnesium, and vitamin D, which persisted when the lowest and the highest deciles of intake were eliminated, but which virtually disappeared when the top 2 and the bottom 2 deciles were dropped from the analyses. These findings suggest a threshold effect, whereby added sugars intake below approximately 19% of calories (high-end decile 8) was not associated with inadequate micronutrient intakes. We also found that even at the lower deciles of added sugars intake, large percentages of the population were below the EAR, particularly for vitamin D. Because of the observation that for certain nutrients children and adolescents in the lowest deciles of added sugars intake had higher prevalences of micronutrient inadequacy than those in the next several higher deciles of intake, these findings may suggest that better nutrient-rich food selection is needed independent of added sugars intake. Overall, our findings suggest that adequate intakes of calcium, magnesium, and vitamin D are difficult to obtain from the US food supply under the current dietary patterns of children and adolescents, regardless of added sugars intake. Consideration of the whole diet, including micronutrient contributions from vitamin and mineral dietary supplements and foods containing various levels of added sugars, would help to inform dietary guidance for children and adolescents. Given recent trends in food product development and reformulation, continued monitoring of added sugars intake and impacts on micronutrient adequacy appears warranted.

## Supplementary Material

nzz126_Supplement_Tables

## Notes

Supported by The Sugar Association, Inc.

Author Disclosures: VLF serves as Vice President of Nutrition Impact. LLC conducts NHANES analyses for numerous members of the food, beverage, and dietary supplement industry. PCG is employed by The Sugar Association, Inc., and garnered funding of the study. MOS is a contract employee with The Sugar Association, Inc. LR and LD as independent consultants provide nutrition and regulatory consulting to various food manufacturers, commodity groups, and health organizations. The views expressed in the manuscript are those of the authors and do not necessarily reflect the position or policy of The Sugar Association, Inc.

Supplemental Tables 1–3 are available from the “Supplementary data” link in the online posting of the article and from the same link in the online table of contents at https://academic.oup.com/cdn/.

Abbreviations used: DGA, Dietary Guidelines for Americans; EAR, estimated average requirement; FPED, Food Patterns Equivalent Database; NCI, National Cancer Institute; RTEC, ready-to-eat cereal; UI, usual intake.
